# Relation of ABO Blood Groups to the Plaque Characteristic of Coronary Atherosclerosis

**DOI:** 10.1155/2017/2674726

**Published:** 2017-11-08

**Authors:** Xingtao Huang, Yongpeng Zou, Lulu Li, Shuyuan Chen, Jingbo Hou, Bo Yu

**Affiliations:** Department of Cardiology, The Second Affiliated Hospital of Harbin Medical University, The Key Laboratory of Myocardial Ischemia, Chinese Ministry of Education, Harbin, Heilongjiang, China

## Abstract

The ABO blood types related to morphological characteristics of atherosclerosis plaque are not clear. We aimed to evaluate the relationship between ABO blood groups and the coronary plaque characteristic. We retrospectively identified the target lesions in 392 acute coronary syndrome patients who underwent optical coherence tomography examination before stenting. Subjects were divided into different groups according to different blood types. The fibrous cap thickness was significantly thicker in O type compared with non-O type (0.075 ± 0.033 mm versus 0.061 ± 0.024, *p* < 0.001). Meanwhile, the incidence of thin-cap fibroatheroma was also significantly higher in O type compared with non-O type (51.0% versus 71.5%, *p* < 0.001). The O type showed a significantly larger minimum lumen area [1.26 (0.82, 2.13) versus 1.05 (0.67, 1.82), *p* = 0.020] and minimum lumen diameter [1.03 (0.74, 1.31) versus 0.95 (0.66, 1.25), *p* = 0.039] compared with non-O type. There were no differences found in incidence of lipid plaque, plaque rupture, and thrombus between different blood type groups even between O type and non-O type group (*p* > 0.05). The plaques of O type blood group were exhibited more stably compared with non-O type blood group. Moreover, the non-O type blood group have more serious coronary artery stenosis than O type blood group.

## 1. Introduction

The ABO histoblood group is one of the major human blood antigen systems, classified by the presence of A and B antigens on the surface of red blood cells. Previous studies have shown that ABO blood groups, particularly the non-O blood groups, are associated with risk factor of coronary artery disease (CAD) [1–3]. Recent metastudy also confirmed that non-O blood group appears to be an independent risk factor for CAD and myocardial infarction, and non-O blood group was associated with a statistically significant 14% increase in CAD incidence relative to O blood group [4]. Moreover, clinical data on the association of ABO blood groups with severity of CAD have shown that ABO blood groups were significantly related to severity of CAD, non-O blood group was found to be the independent predictors of high SYNTAX score [5], and O blood group was shown as a protective factor of high Gensini score [6].

However, the underlying mechanisms for the relationship between ABO blood groups and CAD were unclear. An association of ABO blood group with total cholesterol and low-density lipoprotein cholesterol has long been recognized [7] and non-O blood group have higher cholesterol absorption rates [8] and higher levels of total cholesterol and low-density lipoprotein cholesterol [9]. We hypothesized that the high level of cholesterol in non-O blood increased the risk of CAD and MI by playing an essential role in the progress of atherosclerotic plaque.

Optical coherence tomography (OCT) has been widely used for coronary plaque characterization [10, 11]. In this study, we aimed to analyze OCT images of coronary artery plaque in CAD patients and compare the morphological characteristics between different blood type groups.

## 2. Materials and Methods

### 2.1. Study Design and Population

Our OCT registry is one of multicenter registries of patients who underwent OCT of the coronary arteries which involves 20 sites across 6 countries (The Massachusetts General Hospital OCT Registry, registered on ClinicalTrials.gov, NCT01110538). This was a retrospective observation study. Study subjects were collected from our OCT registry. The subjects with poor OCT image quality were excluded. The detailed demographic, clinical, and hematologic data were collected from all subjects. Subjects were divided into 4 groups according different blood types: A type group, B type group, AB type group, and O type blood group. Further analysis was performed between O type and non-O type. This protocol was approved by the Harbin Medical University Ethics Committee, and all patients received statins and antiplatelet therapy.

### 2.2. OCT Examination and Images Analysis

The time-domain OCT system (M2/M3 Cardiology Imaging System; LightLab Imaging, Inc., Westford, Massachusetts) or the frequency-domain OCT system (C7-XR OCT Intravascular Imaging System; St. Jude Medical, St. Paul, Minnesota) was used in this study. The methods of intracoronary OCT imaging have been described previously [12]. The OCT images of target lesions were analyzed by two independent investigators. If there was discordance in interpretations between the observers, a consensus reading was obtained by corresponding author. Cross-sectional OCT images were analyzed at 1 mm intervals along the pullbacks. Cross-sectional images were screened for quality assessment. If any portion of the image was outside the screen, if a side branch occupied >45° of the cross-section, or if the image was of poor quality due to residual blood, sew-up artifact, or reverberation, then it was excluded from the analysis [13]. Plaques were classified into 2 categories: (1) fibrous (homogeneous high signal region) or (2) lipid plaque (low signal region with diffuse border). For lipid plaque, the lipid arc was measured at every 1 mm interval throughout the entire length of lipid length. The fibrous cap thickness (FCT) of lipid-rich plaque was measured at its thinnest part 3 times, and the average value was then calculated. Thin-cap fibroatheroma (TCFA) was defined as the thinnest fibrous cap thickness ≤65 *μ*m in lipid-rich plaque on a cross-sectional image [14]. Plaque rupture was identified by the presence of disrupted fibrous cap [14]. Intracoronary thrombus was defined as a mass (diameter ≥ 250 *μ*m) attached to luminal surface or floating within the lumen [12].

### 2.3. Statistics

All statistical analysis was performed by an independent statistician. Categorical data are presented as frequencies (percentages) and were compared using *χ*^2^ test. Continuous measurements are presented as mean ± SD or median (25th to 75th percentiles) depending on the normality of data. Student's *t*-test and analysis of variance (ANOVA) were used to compare the means of the continuous measurements while the nonparametric test was used to compare the median of the continuous measurements. All analysis was performed using SPSS 23 (SPSS, Inc., Chicago, Illinois). For the multiple comparisons among the 4 groups, Bonferroni's correction was applied; thus, a *p* value < 0.008 was considered statistically significant. For other comparisons a *p* value < 0.05 was considered to represent a statistically significant result.

## 3. Results

### 3.1. Patients Characteristics

Totally 392 subjects were included in this study. The distribution of patients was 115 (29.3%) in A type group, 128 (32.7%) in B type group, 39 (9.9%) in AB type group, and 110 (28.1%) in O type group. There are no differences in clinical characteristics among different blood groups. The baseline clinical characteristics were shown in Table 1.

### 3.2. OCT Findings

**Figure 1:**
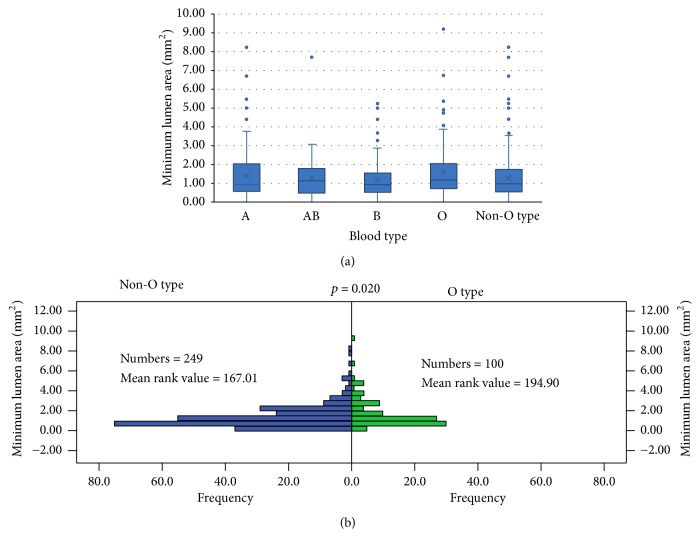
*The minimum lumen area in different blood type subjects evaluated by optical coherence tomography*. (a) Box plots represented medians and the 25%ile and 75%ile as boxes and the 2.5%ile and 97.5%ile as whiskers. The minimum lumen area was larger in O type subjects compared with non-O type subjects (*p* = 0.020). There was no significant difference among A type, AB type, B type, and O type subjects (*p* = 0.077). (b) The frequency distribution of minimum lumen area in O type subjects and non-O type subjects.

**Figure 2:**
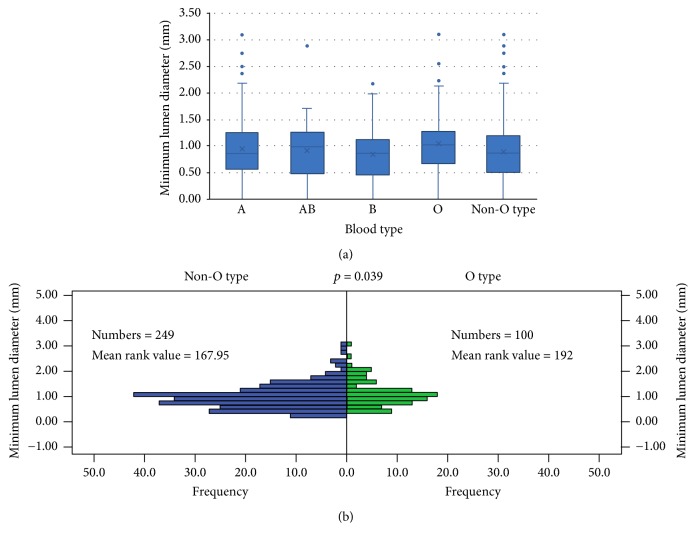
*The minimum lumen diameter in different blood type group evaluated by optical coherence tomography.* (a) Box plots represented medians and the 25%ile and 75%ile as boxes and the 2.5%ile and 97.5%ile as whiskers. The minimum lumen diameter was larger in O type subjects compared with non-O type subjects (*p* = 0.039). There was no significant difference among A type, AB type, B type, and O type subjects (*p* = 0.070). (b) The frequency distribution of minimum lumen diameter in O type subjects and non-O type subjects.

**Figure 3:**
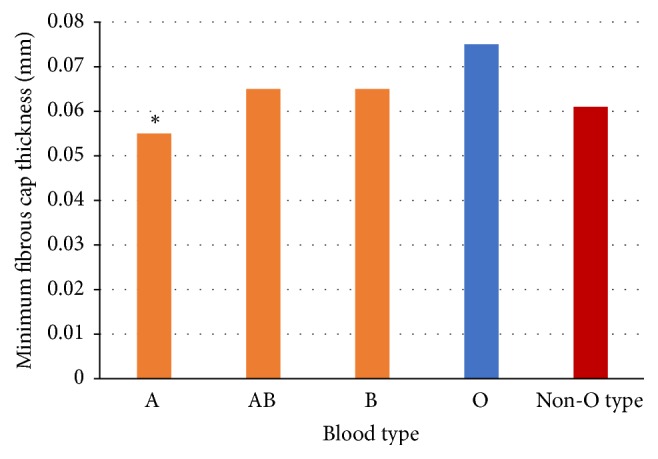
*The minimum FCT in different blood type group evaluated by optical coherence tomography.* Compared to non-O type, the O type subjects showed a thicker minimum fibrous cap thickness (0.075 ± 0.033 versus 0.061 ± 0.024, *p* < 0.001). There was no significant difference found in AB type subjects and O type group after adjustment by Bonferroni's correction (*p* value < 0.008 was considered statistically significant), between neither B type nor O type. ^*∗*^The A type subjects showed a significantly thinner minimum fibrous cap thickness compared with O type subjects (*p* < 0.001).

**Figure 4:**
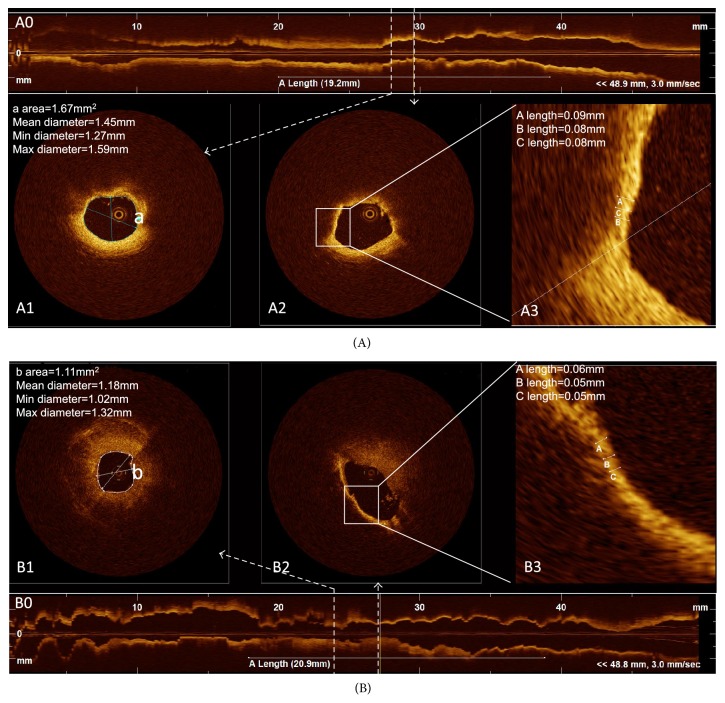
*Representative images for optical coherence tomography analysis*. (A) Representative optical coherence tomography (OCT) images for O type subjects. (B) Representative OCT images for non-O type subjects. A0 and B0 show longitudinal section of vessel and the length of white line is the length of plaque. A1 and B1 show the cross-section image of minimum lumen. A2 and B2 show the cross-section image of lipid-rich plaques with minimum fibrous cap thickness. A3 and B3 have enlarged the measurement site of fibrous cap thickness.

The OCT results were shown in Table 2. Compared to non-O type subjects, the O type subjects had a thicker minimum FCT, lower incidence of TCFA, larger minimum lumen area (Figure 1), and minimum lumen diameter (Figure 2), although there was no difference in minimum lumen area (*p* = 0.077) and minimum lumen diameter (*p* = 0.070) among different blood groups. The O type subjects showed a thicker minimum FCT compared to other blood subjects (A, AB, and B type subjects); however, a statistically significant difference (*p* < 0.001) was only found in the comparison between O type subjects and A type subjects after Bonferroni's correction was applied (Figure 3). There were no differences found in incidence of lipid plaque, plaque rupture, and thrombus between different blood type subjects even between O type and non-O type subjects (*p* > 0.05). The mean lipid core arc also had no significant difference among different blood subjects (*p* > 0.05). Representative images for OCT analysis are shown in Figure 4.

## 4. Discussion

To the best of our knowledge, this study firstly prospectively examined the coronary plaque characteristic in different blood type. The main findings of our study are that (1) O type subjects have thicker FCT and less TCFA compared to non-O type subjects and (2) O type subjects have larger minimum lumen area and minimum lumen diameter compared to non-O type subjects.

It is known that FCT is the most critical component of plaque stability. Thinner caps are more prone to rupture than thicker caps, and the threshold of 65 *μ*m has been widely adopted to identify high-risk lesions [15]. A plaque with a FCT less than 65 *μ*m, with or without previous plaque rupture is considered to be TCFA, which is one indication of a vulnerable plaque. It is also known that the plasma hs-CRP concentration has been proved to be the strongest independent predictor of TCFA and have an inverse linear correlation with FCT [16]. Previous study has reported that patients with O type had lower high-sensitivity C-reactive protein (hs-CRP) levels compared with that of non-O [17]. Therefore, we assume that thicker FCT found in O type subjects in our study may be due to the lower hs-CRP levels. Further study is needed to evaluate the relationship between FCT and blood types.

Subjects of non-O type had been proved with higher levels of total cholesterol, low-density lipoprotein cholesterol, and non-high-density lipoprotein cholesterol compared with that in O type [9]. The levels of cholesterol are related to the severity of coronary artery disease and the atherosclerotic plaque size [18, 19]. This may be one of the mechanism for the minor lumen area in non-O type subjects compared to O type subjects in this study. Because all the patients have received the statins therapy since admitted to hospital, there were no significant differences found in the levels of cholesterol between different two groups due to the lipid-lowering effect of statins in this study. Previous study also found that group O was a protective factor for midhigh Gensini score of coronary atherosclerosis [6]. Our study first time confirmed the relationship between O type blood and the severity of coronary atherosclerosis.

Several studies have reported a significant relationship between ABO blood groups and acute coronary events, especially acute myocardial infarction and sudden cardiac death [20–22]. A consistent relation between non-O blood group and an increased CHD risk was also observed in cross-sectional case-control studies [23–25], while data from prospective cohort studies were inconsistent [3, 7, 26–28]. In this study a significant relationship between ABO blood groups and CHD was also found, but only in plaque characteristic not in CHD risk and acute coronary events, probably because of the patient selection bias. The patients selected for our study were all in sick (with CAD), which was unable to evaluate the relationship between ABO blood groups and CHD risk. Furthermore, most of patients in this study had been evaluated for the risk for OCT imaging examination and the patients in high procedure risk and the patients with poor OCT image quality were excluded, both of which would cause patient selection bias. The same reason may explain why no statistical difference was found in plaque rupture while a significant difference was found in TCFA between non-O type group and O-type group in this study.

The present study has several limitations. This study is a retrospective observational study from a registry database and, therefore, limited by the possibility of selection bias inherent to retrospective studies (as mention above). Lack of follow-up data also is a limitation of this study; long-term follow-up is needed to evaluate the clinical outcome of different blood type groups. In addition, the sample of this study was limited to evaluate the correlation between ABO blood groups and CHD risk; larger sample study is needed.

## 5. Conclusions

In conclusion, a significant relationship between O-type blood and plaque characteristic of coronary arteries atherosclerosis is observed. The plaques of O type blood group were exhibited more stably compared with non-O type blood group. Moreover, the non-O type blood group have more serious coronary artery stenosis than O type blood group.

## Figures and Tables

**Table 1:** Clinical characteristics.

Characteristics	Non-O	O	A	B	AB	*p* ^*∗*^	*p* ^#^
Patients number	282	110	115	128	39	—	—
Male, *n* (%)	210 (74.5%)	84 (73.6%)	79 (68.7%)	103 (80.5%)	28 (71.8%)	0.205	0.866
Age, year	57.81 ± 10.73	58.64 ± 8.83	59.52 ± 10.48	55.57 ± 11.59	59.80 ± 7.71	0.088	0.492
Smoke, *n* (%)	152 (53.9%)	57 (51.8%)	66 (57.4%)	68 (53.1%)	18 (46.2%)	0.643	0.710
Hypertension, *n* (%)	157 (55.7%)	61 (55.5%)	71 (61.7%)	66 (51.6%)	20 (51.3%)	0.408	0.969
Diabetes Mellitus, *n* (%)	83 (29.4%)	37 (33.6%)	41 (35.7%)	32 (25.0%)	10 (25.6%)	0.241	0.417
Total cholesterol (mmol/L)	4.76 ± 1.26	4.78 ± 1.23	4.79 ± 1.17	4.85 ± 1.40	4.41 ± 1.00	0.300	0.708
Triacylglycerol (mmol/L)	2.28 ± 1.75	2.48 ± 2.25	2.39 ± 1.95	2.32 ± 1.75	2.18 ± 1.79	0.463	0.308
High-density lipoprotein-cholesterol (mmol/L)	1.32 ± 0.47	1.29 ± 0.36	1.30 ± 0.38	1.38 ± 0.54	1.30 ± 0.35	0.226	0.218
Low-density lipoprotein cholesterol (mmol/L)	2.31 ± 0.88	2.29 ± 0.75	2.35 ± 0.86	2.43 ± 0.92	2.01 ± 0.68	0.188	0.642
STEMI, *n* (%)	78 (27.7%)	29 (26.4%)	29 (25.2%)	44 (34.4%)	5 (12.8%)	0.087	0.430
NSTE-ACS, *n* (%)	149 (52.8%)	65 (59.1%)	61 (53.0%)	65 (50.8%)	23 (59.0%)		
SA, *n* (%)	55 (19.5%)	16 (14.5%)	25 (21.7%)	19 (14.8%)	11 (28.2%)		

STEMI = ST segment elevation myocardial infarction, NSTE-ACS = no ST segment elevation acute coronary syndrome, and SA = stable angina. ^*∗*^*p* value for comparison among O, A, B, and AB type groups. ^#^*p* value for O versus non-O type groups.

**Table 2:** The plaque characteristics of target lesion by OCT imaging.

Characteristics	Non-O	O	A	B	AB	*p* ^*∗*^	*p* ^#^
Total lesions, *n*	282	110	115	128	39	—	—
Lipid plaque, *n* (%)	249 (88.3%)	100 (90.9%)	105 (91.3%)	112 (87.5%)	32 (82.1%)	0.353	0.457
Lipid rich plaque	247 (99.2%)	98 (98.0%)	105 (100%)	111 (99.1%)	31 (36.9%)	0.355	0.324
Minimum FCT, mm	0.061 ± 0.024	0.075 ± 0.033	0.055 ± 0.019	0.065 ± 0.027	0.065 ± 0.022	<0.001	<0.001
TCFA, *n* (%)	178 (71.5%)	51 (51.0%)	86 (81.9%)	70 (62.5%)	22 (68.8%)	<0.001	<0.001
Plaque rupture, *n* (%)	112 (39.7%)	40 (36.4%)	49 (42.6%)	53 (41.4%)	10 (25.6%)	0.242	0.540
Mean lipid core arc	219.78 ± 56.18	214.51 ± 58.27	222.20 ± 55.34	215.64 ± 52.70	226.38 ± 70.04	0.609	0.433
Thrombosis, *n* (%)	88 (31.2%)	34 (30.9%)	34 (29.6%)	45 (35.2%)	9 (23.1%)	0.516	0.955
Minimum lumen diameter, mm	0.95 (0.66, 1.25)	1.03 (0.74, 1.31)	0.94 (0.65, 1.28)	0.94 (0.63, 1.16)	1.06 (0.80, 1.33)	0.070	0.039
Minimum lumen area, mm^2^	1.05 (0.67, 1.82)	1.26 (0.82, 2.13)	1.01 (0.68, 2.08)	1.03 (0.63, 1.67)	1.20 (0.75, 1.97)	0.077	0.020

FCT = fibrous cap thickness. TCFA = thin-cap fibroatheroma. ^*∗*^*p* value for comparison among O, A, B, and AB type groups. ^#^*p* value for O versus non-O type groups.
